# Notes on the genus *Xestopus* from China, with description of a new species (Carabidae,Sphodrini, Dolichina)

**DOI:** 10.3897/zookeys.1009.61515

**Published:** 2021-01-11

**Authors:** Pingzhou Zhu, David H. Kavanaugh, Hongbin Liang

**Affiliations:** 1 Key Laboratory of Zoological Systematics and Evolution, Institute of Zoology, Chinese Academy of Sciences, Beijing 100101, China Institute of Zoology, Chinese Academy of Sciences Beijing China; 2 College of Life Science, University of Chinese Academy of Sciences, Beijing 100049, China University of Chinese Academy of Sciences Beijing China; 3 Department of Entomology, California Academy of Sciences, Golden Gate Park, San Francisco, CA 94118, USA Department of Entomology, California Academy of Sciences San Francisco United States of America; 4 Research Professor, Biology, San Francisco State University, San Francisco, California, USA San Francisco State University San Francisco United States of America

**Keywords:** China, Dolichina, endophallus, key, new species, *
Xestopus
*

## Abstract

The genus *Xestopus* Andrewes, 1937 in China is reviewed, with the description of a new species: *X. gutangensis* Zhu & Kavanaugh, **sp. nov.** (type locality: Xizang: Mêdog, 29.46414°N, 95.73563°E). The male of *X. cyaneus* Sciaky & Facchini, 1997 is described for the first time, and the first record of this species in Yunnan, China, represents an eastward range extension for the species. A key is provided for the eight known species of the genus.

## Introduction

*Xestopus* Andrewes, 1937 (Sphodrini, Dolichina) is a small genus previously comprised of seven species, all distributed along the Himalaya (Nepal, Bhutan, China, and Myanmar). This genus is differentiated from related genera mainly by its larger size (15 mm > BL > 25 mm) and the hooked right paramere of the male genitalia (Sciaky and Wrase 1998).

Before the present study, only one species, *Xestopus cyaneus* Sciaky & Facchini, 1997, had been recorded from China. This species is distinct among *Xestopus* species, with its bluish elytra, absence of the anterior pair of supraorbital setae, full-sized hind wings, and smaller size (16 mm) (Sciaky and Facchini 1997). In recent expeditions to Xizang, China, specimens of *X. cyaneus* and a new species were collected. The new species is very similar to *X. cyaneus*, except for the presence of the anterior pair of supraorbital setae.

In this article, we (1) describe the new species, (2) provide additional morphological data for *X. cyaneus*, including the first descriptions of male and female genitalia, (3) discuss previous erroneous distributional records, and (4) provide a revised key to all known species of genus *Xestopus*.

## Materials and methods

Specimens examined during our study are deposited in the following collections:

**CAS**California Academy of Science, San Francisco, USA;

**CCCC** Collection of Changchin Chen, Tianjin, China;

**CRS** Collection of Riccardo Sciaky, Milano, Italy;

**IZAS**Institute of Zoology, Chinese Academy of Sciences, Beijing, China;

**NZSI**Zoological Survey of India, National Zoological Collection, Calcutta, India.

Abbreviations for measurements used in the paper are as follows: body length (**BL**) was measured from the apical margin of the labrum to the elytral apex; body width (**BW**) was measured across the elytral greatest width (**EW**). Pronotum width (**PW**) was measured across its greatest width; basal width (**PBW**) was measured along its basal margin; apical width (**PAW**) was measured between the apices of the anterior angle, pronotum length (**PL**) was measured along its median line. Elytra length (**EL**) was measured along the suture from the base of the scutellum to the elytra apex. The methods of dissection, illustrations, and measurements mainly follow our previous work (Shi et al. 2013; Shi and Liang 2015).

## Taxonomy

### Genus Xestopus

Taxon classificationAnimaliaColeopteraCarabidae

Andrewes, 1937

2AACC6DF-9C28-5942-89C2-5E4E529A077F

Andrewes 1937: 59; Morvan 1979: 41 (mentioned only); Casale 1981: 389 (key to species); Morvan 1982: 45 (described new species); Casale 1988: 138 (placed in Dolichina); Sciaky and Facchini 1997: 235 (described new species); Sciaky and Wrase 1998: 223 (key to genera of Dolichina); Lorenz 1998: 373 (catalogue); Hovorka and Sciaky 2003: 530 (catalogue); Lorenz 2005: 399 (catalogue); Hovorka 2017: 769 (catalogue); Schmidt and Will 2020: 336 (diagnosis).
Synonym: Nepalocalathus Habu, 1973: 100, type species Calathus kumatai Habu, 1973; Habu 1978: 302 (raised to genus rank and transferred to Dolichina); Casale 1981: 389 (synonymized with Xestopus).
Synonym: Wittmerosphodrus Morvan, 1978: 100, type species Wittmerosphodrus walteri Morvan, 1978; Morvan 1979: 36 (described a new species); Casale 1981: 389 (synonymized with Xestopus).

#### Type species.

*Pristonychus alticola* Fairmaire, 1889 (type locality: Mountain Yeomatong, North Myanmar; holotype in NZSI), by monotypy.

#### Diagnosis.

Among the seven genera of Dolichina, *Xestopus* can be distinguished from others by the following character combination: third antennomere very long, usually longer than the first two antennomeres combined; tooth of mentum bifid; pronotum more or less cordiform; elytra with microsculpture nearly isodiametric, parascutellar seta present, elytral disc without setigerous pores; lateral grooves absent on metatarsomeres I–IV; males with right paramere hooked at apex; female apical gonocoxite with one ensiform seta at external margin (in most specimens) and sensory pit absent. Detailed descriptions and a key to the genera in the subtribe Dolichina have been provided by Casale (1981) and Sciaky and Wrase (1998).

#### Comparisons.

This genus is most similar to the genus *Dolichus* Bonelli, sharing the large body size (>15 mm), but its members differ from those of the latter in having the tooth of the mentum bifid, the pronotum cordiform, elytral interval 3 without setigerous pores, and the right paramere of male genitalia apically hooked.

#### Distribution.

This genus includes eight species distributed along the Himalayas (two in Nepal, three in Bhutan, two in China, and one in Myanmar).

#### Remarks.

The type species of this genus, *Xestopus alticola* (Fairmaire, 1889) was described from Mount Yeomatong, northern Myanmar. In the two versions of the *Catalogue of Palearctic Coleoptera* (Hovorka and Sciaky 2003; Hovorka 2017), the distribution of this species includes Sichuan and Sikkim and both these two are doubtful. The record for Sikkim was added by Andrewes (1937); however, this locality and the type locality are extremely distant from each other for a species with apterous members. In addition, the record for Sikkim maybe a different species (Morvan 1979; Casale 1981). No other literature records have reported *X. alticola* from Sichuan, and, in fact, no *Xestopus* specimens have been found in that province during our many expeditions in Sichuan, so we also doubt the reliability of this distributional record.

Casale (1981) pointed out that the three species from Bhutan (*X. walteri* (Morvan, 1978), *X. bhutanensis* (Morvan, 1979), and *X. cordicollis* (Morvan, 1979)) may represent three subspecies of a single species or eventually be combined into one species when abundant materials are available. Conversely, the male allotype and female holotype of *X. nepalensis* probably represent two different species, in our opinion, based on the original description and figures. The male differs from the female in having elytra with (1) rufous color in the scutellar region and along the sutural margin to mid-length, (2) the humeri more rounded, (3) the basal margination markedly sinuate, and (4) the apices more rounded and slightly oblique medially. Thus, a revision appears necessary to deal with this situation.

### Xestopus gutangensis

Taxon classificationAnimaliaColeopteraCarabidae

Zhu & Kavanaugh sp. nov.

73AC6D00-B435-5132-BACC-7E915B88F3B0

http://zoobank.org/9BF90A02-37CD-4FF6-B5A2-5EF5F4C90A3D

Figures 1 , 3–7 , 8–10 , 19

#### Type locality.

China, Xizang: Mêdog (29.46414°N, 95.73563°E), altitude 2025 m.

#### Type material.

***Holotype*.** Мale (IZAS), body length = 16.9 mm, board mounted, genitalia dissected and glued on plastic film pinned under specimen, “Xizang, Nyingchi Prefecture, Mêdog County, Gutang Township, Xingkai village, 29.46414°N, 95.73563°E”; “2025 m, 2019.VIII.15 N, Liang H.B. & Xu Y. lgt., Institute of Zoology, IZAS”; “HOLOTYPE ♂ *Xestopus gutangensis* sp. nov. des. ZHU & KAVANAUGH 2020” [red label]. ***Paratypes*** (34 males and 45 females): one female (IZAS), “CHINA, Yunnan, Gongshan County, Dulongjiang Township, Bapo, 1412 m, 27.73902°N, 098.34975°E, 26 October 2004, Stop # LHB-2004-033, H.-B. Liang collector”; 14 males and 13 females (CAS, IZAS), “CHINA, Yunnan, Gongshan County, Dulongjiang Township, Bapo, Mulangdang, 1355 m, 27.75256°N, 098.34745°E, 4 November 2004, Stop # LHB-2004-046, H.-B. Liang collector”; one male and three females (CAS, IZAS), “CHINA, Yunnan, Gongshan County, Dulongjiang Township, 0.6 km N of Dizhengdang village on Dulong Jiang, 28.08442°N, 098.32652°E, 1880 m, 29 October 2004, Stop # DHK-2004-061B, D.H. Kavanaugh, G. Tang & D.-Z. Dong collectors”; three females (CAS, IZAS), “CHINA, Yunnan, Gongshan County, Dulongjiang Township, 0.6 km N of Dizhengdang village on Dulong Jiang, 28.08442°N, 098.32652°E, 1880 m, 30 October 2004, Stop # DHK-2004-064, D.H. Kavanaugh, G. Tang & D.-Z. Dong collectors”; one male and three females (CAS, IZAS), “CHINA, Yunnan, Gongshan County, Dulongjiang Township, west bank of Dulong Jiang at Elideng village, 1640 m, 28.00287°N, 098.32145°E, 3 November 2004, Stop # DHK-2004-073, D.H. Kavanaugh, G. Tang & D.-Z. Dong collectors”; one male (CAS), “CHINA, Yunnan, Gongshan County, Dulongjiang Township, 0.5 km N of Kongdang, 1500 m, 27.88111°N, 098.34063°E, 25 October 2004, Stop # DHK-2004-057B, D.H. Kavanaugh , H.-B. Liang & D.-Z. Dong collectors”; two males and three females (CAS, IZAS), “CHINA, Yunnan, Gongshan County, Dulongjiang Township, 0.5 km N of Kongdang, 1500 m, 27.88111°N, 098.34063°E, 25 October 2004, Stop # DHK-2004-057C, D.H. Kavanaugh, Q.-B. Hou, H.-B. Liang, D.-Z. Dong & G. Tang collectors”; six males and eight females (CAS, IZAS), “CHINA, Yunnan, Gongshan County, Dulongjiang Township, Dulong Jiang at Xianjiudang village, 1580 m, 27.94092°N, 098.33340°E, 4 November 2004, Stop # DHK-2004-074, D.H. Kavanaugh, M.A. Dixon, G. Tang & D.-Z. Dong collectors”; one male and five females (IZAS), the same collecting data as holotype; six males and four females (IZAS, CAS), “CHINA, Tibet, Bomi, Yi’ong, Tangmai bridge, Beach of Yi’ong Zangbo, 30.09633°N, 95.06577°E”; “2035 m, 2006.8.30 N, Liang H.B., Song Z.S., Institute of Zoology, Chinese Acad. of Sciences”; one male and one female (IZAS), “CHINA, Xizang, Nyingchi, Bomi, Yi’ong, Tangmai bridge, Beach of Yi’ong Zangbo, 30.09633°N, 95.06577°E”; “2035m, 2020.VIII.31 N, Liang H.B. & Zhang N. lgt., Institute of Zoology, Chinese Acad. of Sciences”; one male and one female (IZAS), “CHINA, Xizang, Nyingchi, Bomi, Yi’ong, Tangmai bridge, Beach of Yi’ong Zangbo, 30.09633°N, 95.06577°E”; “2035 m, 2020.IX.1 N, Liang H.B. & Zhang N. lgt., Institute of Zoology, Chinese Acad. of Sciences”; All paratypes also bear the following label: “PARATYPE *Xestopus gutangensis* sp. nov. des. ZHU & KAVANAUGH 2020” [red label].

#### Diagnosis.

Dorsum black, elytra with more or less bluish metallic luster. Anterior supraorbital setae present. Pronotum with lateral margins faintly sinuate before posterior angles. Apices of elytra rounded (Fig. 6). Hind wings full-sized. Metepisternum long and narrow.

#### Comparisons.

This new species is most similar to *Xestopus cyaneus*, sharing the bluish elytra and full-sized hind wings, which are distinctive features in the genus. *X. gutangensis* sp. nov. can be readily distinguished from *X. cyaneus* by: (1) anterior pair of supraorbital setae present; (2) apices of elytra rounded; (3) apical lamella of median lobe strongly bent ventrally like a hook; (4) endophallus without a densely setose area on left side; (5) gonocoxite II of female ovipositor very long. Supernumerary setae have been found in some *X. gutangensis* specimens in the area of the posterior supraorbital setae, on the pronotal lateral margins and posterior angles, and on the external margin of gonocoxite II (as ensiform setae). These additional setae were not observed on any of the specimens of *X. cyaneus* that we examined.

#### Description.

**Figures 1, 2.**
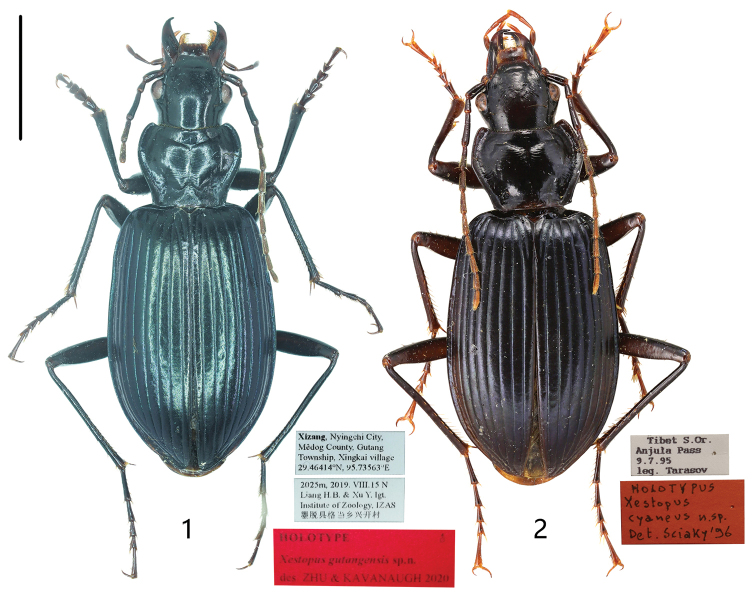
Holotypes of *Xestopus* spp. **1***X. gutangensis* Zhu & Kavanaugh, sp. nov. (male, Xizang, IZAS) **2***X. cyaneus* Sciaky & Facchini, 1997 (female, Xizang, CRS). Scale bar: 5.0 mm.

BL = 15.7–18.0 mm, BW = 6.4–7.8 mm. Dorsum (Fig. 1) black, elytra with more or less bluish metallic luster, very faint in some specimens; appendages dark, antennomeres 4–11, labial and maxillary palpi, apex of mouthparts and tarsomeres dark brown; venter black, without metallic luster. Head, pronotum and elytra with strong isodiametric microsculpture.

***Head*** with vertex smooth; frontal impressions shallow and curved, in front of eyes; clypeus with anterior margin faintly emarginate; labrum with anterior margin straight; temporae slightly swollen behind eyes; both anterior and posterior pairs of supraorbital setae present (two pairs of posterior supraorbital setae present in a few specimens); antennae long and slender, extended to basal one-third of elytra.

***Pronotum*** cordiform, slightly transverse, PW/PL = 1.38–1.55, widest near anterior quarter; anterior margin markedly concave, slightly wider than basal margin, PAW/PBW = 1.13–1.33, lateral margins broadly rounded before middle, then distinctly narrowed to base, faintly sinuate before posterior angles, PW/PBW = 1.31–1.50, lateral margins with one pair of setae at widest points and posterior angles respectively (with an additional seta on one or both sides and at either or both widest points and posterior angles), basal margin straight; anterior angles rounded, moderately extended forward, posterior angles distinctly obtuse; disc glabrous, gently convex; median line fine but clearly defined; basal fovea large and deep, without punctures but with some wrinkles; lateral explanations very wide and impunctate.

***Elytra*** wide, EL/EW = 1.44–1.59, moderately dilated towards apex, widest near posterior third, apices rounded; basal margination complete and straight; humeral angles rounded, without teeth; intervals moderately convex, striae shallow and impunctate; parascutellar striae well developed and short, between suture and stria 1; parascutellar pores present; interval 3 without setigerous pores; umbilicate series on interval 9 composed of approximately 25 setigerous pores, continuous in middle. Hind wings fully sized.

***Venter*.** Propleuron, mesoepisternum, and metepisternum glabrous, metepisternum long and narrow; all abdominal sternites with a few shallow wrinkles laterally.

***Legs*** long and slender, all tarsi smooth, claws distinctly denticulate in basal half.

**Figures 3–18.**
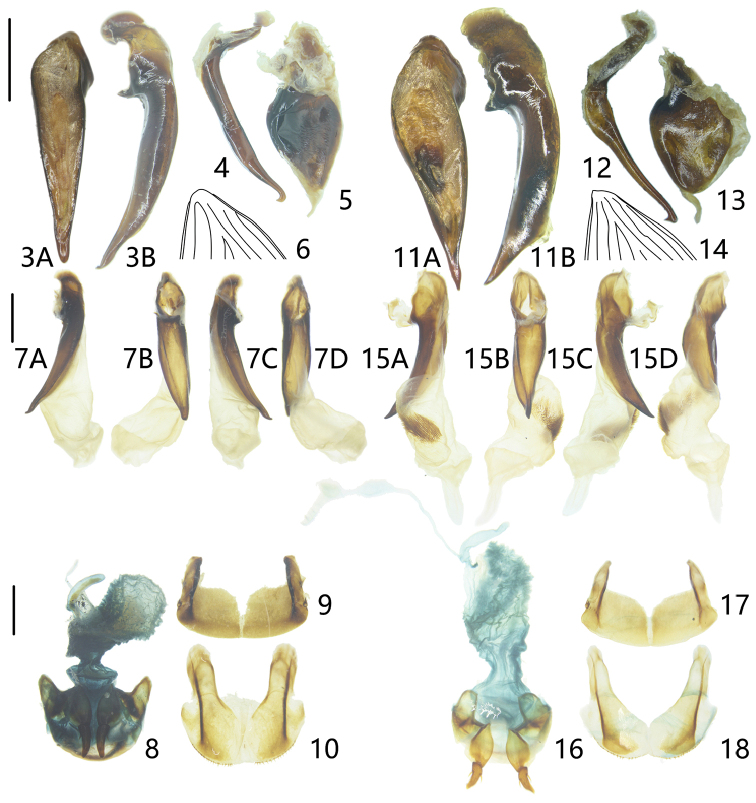
Morphological features of *Xestopus* spp. **3–6** holotype of *X. gutangensis* Zhu & Kavanaugh, sp. nov. **7–10** paratype of *X. gutangensis* Zhu & Kavanaugh, sp. nov. **11–18***X. cyaneus* Sciaky & Facchini, 1997 **3, 11** median lobe of aedeagus **A** dorsal view **B** left lateral view **4, 12** right paramere **5, 13** left paramere **6, 14** apex of left elytron **7, 15** endophallus **A** left lateral view **B** ventral view **C** right lateral view **D** dorsal view **8, 16** female reproductive system **9, 17** female tergum VIII **10, 18** female sternum VIII. Scale bars: 1.0 mm.

***Male genitalia*.** Median lobe (Figs 3, 7) long, slender, and straight but slightly bent ventrally; apical orifice opened dorsally, very long and wide, from basal bulb to apical lamella; in dorsal view (Fig. 3A), left and right margins both straight, apical lamella long, length about twice its basal width, apex rounded; in left lateral view (Fig. 3B), ventral margin weakly expanded in the middle, apical portion slightly bent dorsally and then strongly bent ventrally like a hook at apex. Left paramere (Fig. 5) large and round, with a membranous filament at apex. Right paramere (Fig. 4) markedly styloid and curved, the angle between basal portion and apical portion near 90°; apical portion moderately bent ventrally, apical hook rounded. Endophallus (Fig. 7) simple, with only a single large lobe, straight, extended right at an angle of about 30° relative to longitudinal axis of the median lobe in dorsal view (Fig. 7D); surface smooth, without setae or scales; gonopore and gonopore lobe folded in this specimen.

***Female genitalia*** (Figs 8–10). Gonocoxite II of ovipositor very long, length about four times basal width, with one ensiform seta at external margin in most specimens (two ensiform setae in a few specimens and ensiform seta very small in some specimens), sensory pit of apical gonocoxite absent. Bursa copulatrix very large, rounded. Spermatheca moderately long and tube-like, length about five times maximum width.

#### Distribution

**Figure 19.**
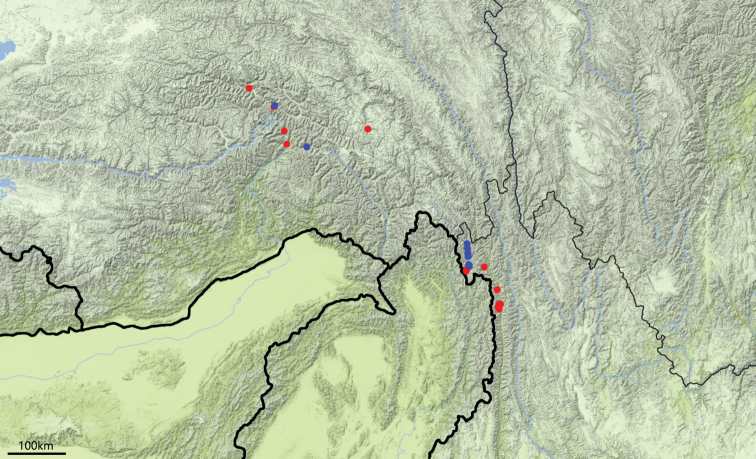
Distribution map for *Xestopus* from China: *X. gutangensis* sp. nov. (blue); *X. cyaneus* (red).

(Fig. 19). This species is known from Bomi and Mêdog counties, Xizang, and from the northern part of the Dulongjiang valley in Gongshan County, Yunnan.

#### Etymology.

The new species is named for Gutang Township, where the type locality, Mêdog, is located.

#### Affinities.

Among all *Xestopus* species, only *X. gutangensis* and *X. cyaneus* have the bluish elytra and full-sized hind wings. Thus, a close relationship of these two species is likely, and the absence of anterior supraorbital setae in the latter is clearly apomorphic within the genus.

### Xestopus cyaneus

Taxon classificationAnimaliaColeopteraCarabidae

Sciaky & Facchini, 1997

4259CDC7-5E6C-5A16-9D17-EF53233310FE

Figures 2 , 11–18 , 19

Xestopus cyaneus Sciaky & Facchini, 1997: 235 (type locality: Anjula Pass, SE Tibet, China; holotype in CRS); Lorenz 1998: 373 (catalogue); Hovorka and Sciaky 2003: 530 (catalogue); Lorenz 2005: 399 (catalogue); Hovorka 2017: 769 (catalogue).

#### Material examined.

Total 196 specimens. ***Holotype*** of *Xestopus cyaneus* Sciaky & Facchini, 1997, by monotypy (CRS): female, body length = 16.1 mm, board mounted, “Tibet S. Or. Anjula Pass 9.7.95 leg. Tarasov”, “HOLOTYPUS Xestopus cyaneus n. sp. Det. Sciaky 1996” [red label]. One male and one female (CRS), “China, Tibet, Motuo co., Hanmi, VI.2013”; one male and one female (CRS), “China, Tibet, Motuo co., Hanmi, VIII.2013”; one male and three females (CRS), “China, Tibet, Linzhi area, Linzhi, 2000 m, VIII–IX.2019”; three females (CRS), “China, Tibet, Linzhi area, Mt. Serkyla, 3000–4000 m, VIII.2018”; four females (CRS), “China, Tibet, Bomi county, 2500 m, VIII.2019”; one female (IZAS), “China, Yunnan, Fugong, Lishadi Town, 4km below Shibali Road, 27.15727°N, 98.79784°E, 2280 m, 2005.VIII.11 N, Tang G lgt.”; two males and three females (CAS, IZAS), “CHINA, Yunnan, Fugong County, Lishadi Township, 4 km E of Shibali on Shibali Road, 2280 m, 27.15727°N, 098.79784°E, 11 August 2005, Stop# DHK-2005-076A, D. Z. Dong collector”; five males and two females (CAS, IZAS), “CHINA, Yunnan, Fugong County, Lishadi Township, Shibali Road from Galadi village to 2.5 km W, 27.13863°N, 098.82174°E to 27.14286°N, 098.82001°E, 1845–1940 m, 9 August 2005, Stop #DHK-2005-071, D.H. Kavanaugh, H.B. Liang, & D. Z. Dong collectors”; one female (IZAS), “CHINA, Yunnan, Fugong County, Lishadi Township, Yamu He at Shikuliudi village, 27.11876°N, 098.83118°E, 1800 m, 26 April 2004, Stop #LHB-2004-008B, Liang H.-B. collector”; one female (CAS), “CHINA, Yunnan, Fugong County, Lumadeng Township, 4 km E of Lao Shibali on Lao Shibali Road, 2120 m, 27.09700°N, 098.80570°E, 21 August 2005, Stop# DHK-2005-101, D. Z. Dong collector”; three males and three females (CAS, IZAS), “CHINA, Yunnan, Fugong County, Lumadeng Township, 1.5 km above confluence of North and South Forks of Yamu He on Lao Shibali Road, 1825 m, 27.11992°N, 098.83150°E, 15 August 2005, at night, Stop# LHB-05-55, H.B. Liang collector”; three females (IZAS), “China, Yunnan, Gongshan, Dulongjiang, Maku village, 27.684453°N, 98.30547°E, 1691 m, 2019. VIII.21 N, Liang HB & Xu Y lgt.”; one female (IZAS), “CHINA, Yunnan, Gongshan County, Dulongjiang Township, Maku, 1823 m, 27.68553°N, 098.30425°E, 2 November 2004, Stop # LHB-2004-042, H.-B. Liang collector”; three males and three females (CAS, IZAS), “CHINA, Yunnan, Gongshan County, Dulongjiang Township, Maku village, 1800 m, 27.68498°N, 098.30299°E, 28 August 2006, Stop #DHK-2006-100, D.H. Kavanaugh, J.A. Miller, & D.Z. Dong collectors”; 43 males and 35 females (CAS, IZAS), “CHINA, Yunnan, Gongshan County, Dulongjiang Township, Maku village, 27.68545°N, 098.30419°E, 1815 m, 2 September 2006, Stop #DHK-2006-119, Y. Liu & D. Z. Dong collectors”; ten males and nine females (CAS, IZAS), “CHINA, Yunnan, Gongshan County, Dulongjiang Township, 0.1 airkm NW of Maku Yakou, 1880 m, 27.67937°N, 098.29617°E, 31 August 2006, Stop #DHK-2006-110, Y. Liu collector”; nine males and seven females (CAS, IZAS), “CHINA, Yunnan, Gongshan County, Dulongjiang Township, 0.5 air km WSW of Maku village on trail to Maku Yakou, 1845 m, 27.68310°N, 098.30038°E, 29 August 2006, Stop #DHK-2006-103, D. H. Kavanaugh collector”; eight males and five females (CAS, IZAS), “CHINA, Yunnan, Gongshan County, Dulongjiang Township, 0.5 air km WSW of Maku village on trail to Maku Yakou, 1845 m, 27.68310°N, 098.30038°E, 29 August 2006, Stop #DHK-2006-108, D.H. Kavanaugh, J.A. Miller, D.Z. Dong, & Y. Liu collectors”; two males (CAS, IZAS), “CHINA, Yunnan, Gongshan County, Dulongjiang Township, 0.5 airkm WSW of Maku village on trail to Maku Yakou, 1845 m, 27.68310°N, 098.30038°E, 29 August 2006, Stop #DHK-2006-116, D. Z. Dong collector”; one female (CAS), “CHINA, Yunnan Province, Gaoligong Shan, Nujiang Prefecture, Nujiang State Nature Reserve, Qiqi area, 10.3 air km W of Gongshan, 27.71542°N, 98.56529°E, 2010 m, 9–14 July 2000, Stop#00-22C, D.H. Kavanaugh, C.E. Griswold, Liang H.-B., D. Ubick, & Dong D.-Z. collectors”; one male (IZAS), “China, Tibet, Bomi, Yi’ong, Tongmai bridge, 30.09633°N, 95.06577°E, 2035m, 2006.VIII.30 N, Liang HB & Song ZS lgt.”; one male (IZAS), “CHINA, Xizang, Nyingchi, Bomi, Yi’ong, Tangmai bridge, Beach of Yi’ong Zangbo, 30.09633°N, 95.06577°E, 2035 m, 2020.VIII.31 N, Liang H.B. & Zhang N. lgt.”; one female (IZAS), “CHINA, Xizang, Nyingchi, Bomi, Yi’ong, Tangmai bridge, Beach of Yi’ong Zangbo, 30.09633°N, 95.06577°E, 2035 m, 2020.IX.1 N, Liang H.B. & Zhang N. lgt.”; one male (IZAS), “China, Tibet, Mêdog, Dagmo road to Gutang, 29.5150°N, 95.4642°E, 1679 m, 2016.VIII.16, Qiu TF lgt.”; one female (IZAS), “China, Xizang, Zhamo-Mêdog road 78K, light trap, 29.66570°N, 95.49577°E, 2104 m, 2017.VIII.15 N, Liang HB lgt.”; three males and three females (IZAS), “China, Xizang, Nyingchi City, Mêdog County, Dagmo Township, Zhamo-Mêdog road 80K, 29.657947°N, 95.489994°E, 2073.40 m, 2020.IX.17 N, Liang H.B. & Xu Y. lgt.”; one male (IZAS), “China, Xizang, Nyingchi City, Mêdog County, Dagmo Township, Zhamo-Mêdog road 80K, 29.657947°N, 95.489994°E, 2073.40 m, 2020.IX.17 N, Zhang Neng lgt.”;one female (CCCC), “China, Xizang, Bomi, Tongmai, 2262 m, 2016.VII.24, Lu YQ lgt.”; two males and two females (CCCC), “China, Xizang, Bomi, Yi’ong, 2380 m, 2016.VII.26, Lu YQ lgt.”; one male and two females (CCCC), “China, Xizang, Mêdog, 80K, 2350 m, 2016.VII.30, Lu YQ lgt.”.

#### Diagnosis.

Dorsum (Fig. 2) black, elytra with more or less bluish metallic luster. Anterior pair of supraorbital setae absent (an anterior supraorbital seta present on one or both sides in a very few specimens). Pronotum with lateral margins very faintly sinuate before posterior angles. Apices of elytra acute (Fig. 12). Hind wings full-sized. Metepisternum long and narrow.

#### Supplementary description.

BL = 15.4–18.6 mm, BW = 6.2–7.4 mm. PW/PL = 1.37–1.54. PW/PBW = 1.40–1.59. PAW/PBW = 1.19–1.44. EL/EW = 1.51–1.68.

***Male genitalia*.** Median lobe (Figs 11, 15) long, stout and straight but slightly bent ventrally; apical orifice broadly open dorsally, very long and wide, from basal bulb to apical lamella; in dorsal view (Fig. 11A), left and right margins both straight, apical lamella very long, length about three times as its basal width, moderately dilated in the middle, apex acute; in left lateral view (Fig. 11B), ventral margin straight, not expanded in the middle, apical portion slightly bent ventrally, apical lamella thick and straight. Left paramere (Fig. 13) large and round, with a membranous filament at apex. Right paramere (Fig. 12) strongly styloid and curved, the angle between basal portion and apical portion nearly 100°; apical portion straight, apical hook acute. Endophallus (Fig. 15) simple, only a single large lobe, straight, extended left at an angle of about 45° relative to longitudinal axis of the median lobe in dorsal view (Fig. 15D); surface with a densely setose area present on left side, without scales; gonopore lobe long and narrow, located at apex of endophallus, gonopore directed toward apex.

***Female genitalia*** (Figs 16–18). Gonocoxite II of ovipositor moderately long, length about three times basal width, with one ensiform seta at external margin, sensory pit of apical gonocoxite absent. Bursa copulatrix large and elongate. Spermatheca moderately long and tube-like, length about five times maximum width.

#### Distribution

(Fig. 19). This species is known from Xizang (Bomi and Mêdog counties) and Yunnan (Gongshan and Fugong, new provincial record). The records from Sichuan cited by Hovorka and Sciaky (2003) and Hovorka (2017) is clearly wrong.

#### Remarks.

The number of pairs of supraorbital setae is a very important character within Carabidae for both classification and systematics. In most members of the tribe Harpalini, only one pair of supraorbital setae is present at a middle position in relation to the diameter of the eyes, while most other ground beetles have two pairs of supraorbital setae. A few non-harpaline carabids lack one pair of supraorbital setae, but the other pair is present either in an anterior or a posterior position relative to the eyes instead of at the middle position as in Harpalinae. It is, therefore, usually easy to determine whether it is the anterior or posterior pair of supraorbital setae that is missing. Variation in this apparently apomorphic feature does not appear to have much value in high-level phylogeny. For example, members of genus *Reflexisphodrus* Casale, 1988 and genus *Eosphodrus* Casale, 1988 (Sphodrini, Sphodrina) lack the posterior pair of supraorbital setae while those of Pterostichus subgenus Unitrichus Sciaky, 1997 (Pterostichini) lack the anterior pair just like most members of *Xestopus cyaneus*. The single species of *Unitrichus*, *Pterostichus platyops* Sciaky, 1997, from Yunnan, China, is distinguished from members of other subgenera of *Pterostichus* by the absence of the anterior pair of supraorbital setae and the presence of many setae at the middle of mentum. We have found a second, undescribed species from Yunnan, China with the similar appearance of *P. platyops* and many setae present at the middle of mentum, but with the anterior pair of supraorbital setae present (unpublished data), as in *Xestopus gutangensis* Zhu & Kavanaugh, sp. nov. These two examples show that differences in the number of supraorbital setae between closely related species is possible, even if it is rarely seen.

In our examination of 260 specimens of *X. cyaneus* and *X. gutangensis* Zhu & Kavanaugh, sp. nov., we found only five that had abnormal numbers of supraorbital setae for their species assignment based on other characters. All were clearly *X. cyaneus* based on the shape of the elytral apex and features of either male or female genitalia. Three of these, including one male from Xizang and one male and one female from Yunnan, had an anterior supraorbital seta present on one side but absent from the other. In addition, we found one female from Xizang and one female from Yunnan that had anterior supraorbital setae present on both sides. Consequently, the presence or absence of anterior supraorbital setae is slightly less reliable for distinguishing these two species than are the shapes of the elytral apices and male and female genitalia.

### Key to species of genus *Xestopus* Andrewes, 1937

**Table:** 

1	Elytra with more or less bluish metallic luster; metepisternum long and narrow; hind wings full-sized	**2**
–	Elytra black or brown, without metallic luster; metepisternum short and wide; hind wings atrophied	**3**
2	Anterior pair of supraorbital setae present; apices of elytra rounded	***X. gutangensis* sp. nov.**
–	Anterior pair of supraorbital setae absent; apices of elytra acute	***X. cyaneus* Sciaky & Facchini, 1997**
3	Pronotum with lateral margins slightly rounded at middle; eyes small; 18 mm	***X. alticola* (Fairmaire, 1889)**
–	Pronotum with lateral margins distinctly rounded at middle; eyes large	**4**
4	Apical lamella of median lobe rounded at tip; 15–18 mm	***X. kumatai* (Habu, 1973)**
–	Apical lamella of median lobe truncated at tip; 18–23 mm	**5**
5	Pronotum with lateral margins markedly sinuate before posterior angles; 20 mm	***X. cordicollis* (Morvan, 1979)**
–	Pronotum with lateral margins faintly sinuated before posterior angles	**6**
6	Apices of elytra rounded or slightly oblique; 18–20 mm	***X. nepalensis* Morvan, 1982**
–	Apices of elytra truncate; 19–23 mm	**7**
7	Apices of elytra markedly truncate; pronotum longer, anterior angles more extended anteriorly, posterior angles more rounded; apical hook of right paramere more developed; apical lamella of median lobe narrower; 20–23 mm	***X. walteri* (Morvan, 1978)**
–	Apices of elytra moderately truncate; pronotum wider, anterior angles less extended anteriorly, posterior angles more acute; apical hook of right paramere less developed; apical lamella of median lobe wider; 19–20 mm	***X. bhutanensis* (Morvan, 1979)**

## Supplementary Material

XML Treatment for Xestopus

XML Treatment for Xestopus gutangensis

XML Treatment for Xestopus cyaneus
